# First record of *Calotesvindumbarbatus* Wagner, Ihlow, Hartmann, Flecks, Schmitz & Böhme, 2021 (Squamata: Agamidae) from China, with revised diagnosis of this species

**DOI:** 10.3897/BDJ.10.e77963

**Published:** 2022-01-31

**Authors:** Shuo Liu, Changsheng Zuo, Fawang Yin, Hong Hui, Dingqi Rao

**Affiliations:** 1 Kunming Natural History Museum of Zoology, Kunming Institute of Zoology, Chinese Academy of Sciences, Kunming, China Kunming Natural History Museum of Zoology, Kunming Institute of Zoology, Chinese Academy of Sciences Kunming China; 2 Yingjiang sub-office, Tongbiguan Provincial Natural Reserve Management and Protection Bureau, Yingjiang, China Yingjiang sub-office, Tongbiguan Provincial Natural Reserve Management and Protection Bureau Yingjiang China; 3 Kunming Institute of Zoology, Chinese Academy of Sciences, Kunming, China Kunming Institute of Zoology, Chinese Academy of Sciences Kunming China

**Keywords:** Agamidae, lizard, mtDNA, Tongbiguan Nature Reserve, western Yunnan

## Abstract

**Background:**

Three new species were recently described from the *Calotesmystaceus* Duméril & Bibron, 1837 complex. Of the three new species, *C.vindumbarbatus* Wagner, Ihlow, Hartmann, Flecks, Schmitz & Böhme, 2021 was known only from northern Myanmar.

**New information:**

Seven specimens of lizard were collected from Tongbiguan Nature Reserve, western Yunnan, China. Phylogenetically, these specimens clustered with the type specimens of *Calotesvindumbarbatus* from Myanmar with strong support and showed inappreciable genetic divergence from the type specimens of *C.vindumbarbatus*. We report the first country record of *C.vindumbarbatus* from China. In addition, a supplementary description, based on the newly-collected specimens and revised diagnosis of this species, was provided.

## Introduction

The spectacularly coloured Blue Forest Lizard *Calotesmystaceus*, originally described from “Pays de Birmans” (= Myanmar), was previously considered to be widely distributed from India, Myanmar, China, Laos, Cambodia, Thailand and Vietnam (Bain and Hurley 2011, Hartmann et al. 2013, Chan-Ard et al. 2015, Das 2015, Pham et al. 2018). Hartmann et al. (2013) described the population in Vietnam as a distinct species *C.bachae* Hartmann, Geissler, Poyarkov, Ihlow, Galoyan, Rödder & Böhme, 2013. Wagner et al. (2021) restricted *C.mystaceus* to southern coastal Myanmar and described three new species occurring in Cambodia, China, Laos, Myanmar, Thailand and India. *Calotesgeissleri* Wagner, Ihlow, Hartmann, Flecks, Schmitz & Böhme, 2021 was known from Myanmar and India; *C.goetzi* Wagner, Ihlow, Hartmann, Flecks, Schmitz & Böhme, 2021 was known from Cambodia, China, Laos, Myanmar and Thailand; and *C.vindumbarbatus* was known only from northern Myanmar.

During our field surveys in western Yunnan, China, from 2018 to 2020, seven specimens of lizard, previously confused with *Calotesmystaceus*, were collected from Tongbiguan Nature Reserve. Detailed morphological comparisons and molecular analysis indicated these specimens to be *C.vindumbarbatus*. Herein, we report this new record for China in detail.

## Materials and methods

### Sampling

**Figure 1.**
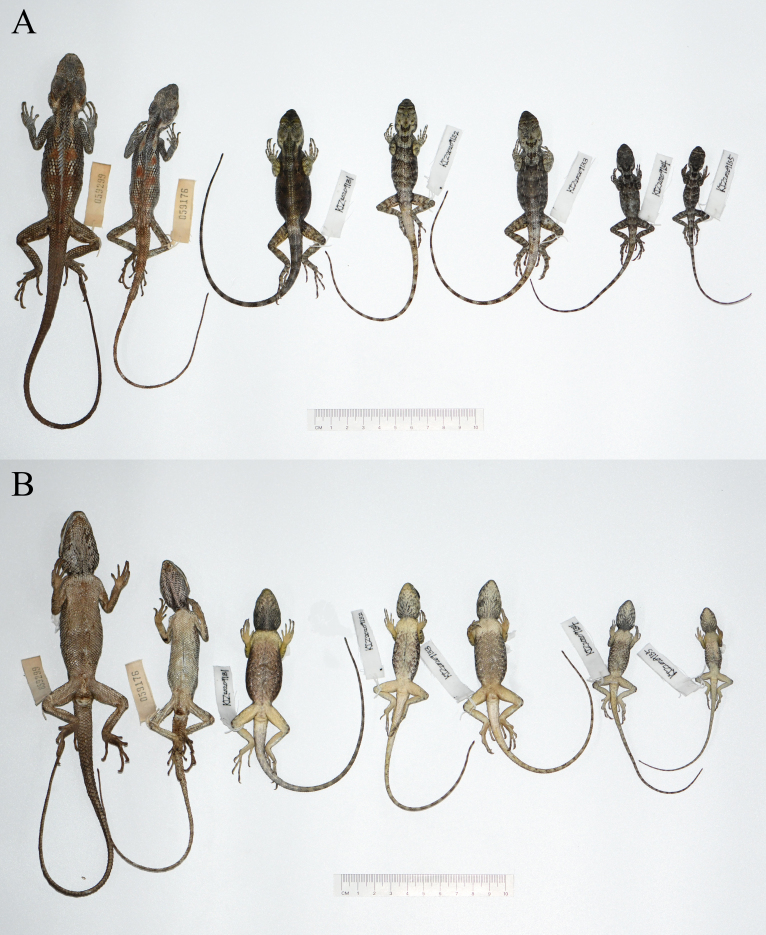
The specimens of *Calotesvindumbarbatus* from Tongbiguan Nature Reserve, western Yunnan, China, in preservative: **A** dorsal view; **B** ventral view.

Field surveys were conducted in Tongbiguan Nature Reserve, Yingjiang County, Dehong Prefecture, Yunnan Province, China, under the permit from the Tongbiguan Provincial Natural Reserve Management and Protection Bureau. Lizards were collected, euthanised and then fixed in 75% ethanol for storage. Liver tissue samples were preserved in 99% ethanol for molecular analysis. The specimens (Fig. 1) were deposited at Kunming Natural History Museum of Zoology, Kunming Institute of Zoology, Chinese Academy of Sciences (KIZ).

### Morphological characteristics

Measurements were taken with a digital caliper to the nearest 0.1 mm, except tail length (TL) which was measured using a string and a ruler. Morphological terminology followed Wagner et al. (2021). Morphometric characters included: 4th finger length (4thFingL), distance from juncture of 3rd and 4th digits to distalmost extent (outer/distalmost surface of claw) of 4th finger; 4th toe length (4thToeL), distance from juncture of 3rd and 4th digits to distal end of 4th digit on hind-foot; Crus length (CrusL), length of tibia from knee to heel; Eye-ear length (EyeEar), distance from anterior edge of tympanum to posterior of orbit (not pupil opening); Forefoot length (ForefL), distance from proximal end of forefoot to tip of fourth digit; Head height (HeadH), dorsoventral distance from top of head to underside of jaw at transverse plane intersecting angle of jaws; Head length (HeadL), distance from anterior edge of tympanum to tip of snout; Head width (HeadW), distance from left to right outer edge of temporal or jaw muscles at their widest point without compression of soft tissue; Hind-foot length (HindfL), distance from proximal end (heel) of hind-foot to distalmost surface of fourth toe; Interorbital width (Interorb), transverse distance between anterodorsal corners of left and right orbits; Jaw width (JawW), distance from left to right outer edge of jaw angles, this measurement excludes jaw musculature broadening of head; Lower arm length (LoArmL), distance from elbow to distal end of wrist or just before underside of forefoot; Naris-eye length (NarEye), distance from anterior edge of orbit to posterior edge of naris; Snout-eye length (SnEye), distance from anterior edge of orbit to tip of snout (rostral scale); Snout-forelimb length (SnForeL), distance from anterior of forelimb, or shoulder, to tip of snout; Snout width (SnW), internasal or internarial distance, transverse distance between left and right nares; Snout-vent length (SVL); tail height (TailH), distance from dorsal to ventral surface of tail base measured just posterior to vent; Tail length (TailL), distance from vent to distal end of tail, noting completeness or regeneration of tail; Tail width (TailW), distance from left to right side of tail base just posterior to vent; Trunk length (TrunkL), body length or axilla-groin length of others, distance between posterior edge of forelimb insertion (axilla) to anterior edge of hindlimb insertion (inguen); Upper arm length (UpArmL), distance from anterior insertion of forelimb, or shoulder, to elbow; Upper leg length (UpLegL), distance from anterior edge of hindlimb insertion to knee. Meristic characters included: Forefoot lamellae (4FingLm), number of 4th digit lamellae, from 1st lamella at digits’ cleft that is wider than deep and touches dorsal digital scale (on at least one side) to most distal lamella, fragmented proximal scales are excluded; Hindfoot lamellae (4ToeLm), analogous to 4FingLm at 4th toe; Canthus rostralis (CanthR), number of elongate scales along ‘dorsolateral snout ridge’ from above posterodorsal corner of nasal scale to and including posteriormost supraciliary scale; Dorsal eyelid scales (Eyelid), number of scales found along dorsal edge of eyelid; Dorsal head scales (HeadSLn), number of scales longitudinally on mid-line between interparietal and rostral scale; Head scales (HeadSTr), number of scales in transverse line between posteriormost left and right supraciliary scales, just anterior of interparietal; Infralabials (Inflab), posterior end defined by posteriormost enlarged scales that touches with Suplab at rear corner of mouth; Mid-body scale rows (MidbS), number of scale rows around trunk at mid-body; Snout scales (SnS), number of scales on line transversally between left and right nasal scales (single scale surrounding naris); Supralabials (Suplab), posterior end defined by posteriormost enlarged scales that touches Inflab at rear corner of mouth; Vertebral scales or spines (VertS), number of mid-dorsal scales (spines or not), beginning with first enlarged spine-like scale on nape to above vent.

### Molecular analysis

Total genomic DNA was extracted from liver tissues with the universal protocol of DNA extraction (Aljanabi and Martinez 1997). A region of mitochondrial genes 12S rRNA was amplified and sequenced by using the primers L1091 (5′–AAAAAGCTTCAAACTGGGATTAGATACCCCACTAT–3′) and H1478 (5′–TGACTGCAGAGGGTGACGGGCGGTGTGT–3′) (Kocher et al. 1989). PCR cycling conditions follow Schmitz et al. (2005) and Nazarov et al. (2012). The products were purified and sequenced by Tsingke Biotechnology (Beijing) Co. Ltd., using the same primers as in PCR. All new sequences were deposited in GenBank. Homologous and outgroup sequences were obtained from GenBank (Table 1).

### Phylogenetic analyses

Sequences were aligned using ClustalW (Thompson et al. 2003) with default parameters in Mega X (Kumar et al. 2018). The genetic distance (uncorrected p-distance) between species was calculated in Mega X (Kumar et al. 2018). The best substitution model GTR+F+I+G4 was selected using the Akaike Information Criterion (AIC) in ModelFinder (Kalyaanamoorthy et al. 2017). Bayesian Inference (BI) was performed in MrBayes 3.2.6 (Ronquist et al. 2012). Two runs were performed simultaneously with four Markov chains starting from the random tree. The chains were run for 1,000,000 generations and sampled every 100 generations. The first 25% of the sampled trees were discarded as burn-in after the standard deviation of split frequencies of the two runs was less than 0.01. The remaining trees were then used to create a consensus tree and to estimate Bayesian posterior probabilities. Maximum Likelihood (ML) analysis was performed in raxmlGUI 2.0 (Silvestro and Michalak 2011) and nodal support values were estimated by 1,000 rapid bootstrap replicates.

## Taxon treatments

### Calotes vindumbarbatus

Wagner, Ihlow, Hartmann, Flecks, Schmitz & Böhme, 2021

A540C62E-C105-57B7-A108-C0F7E17E15C5

#### Materials

- **Type status:** Other material. **Occurrence:** catalogNumber: KIZ 059299; individualCount: 1; sex: male; lifeStage: adult; **Location:** country: China; stateProvince: Yunnan; locality: Nabang Town, Yingjiang County, Dehong Prefecture; verbatimElevation: 320 m; verbatimCoordinates: 24°45'47"N 97°34'15"E; **Event:** eventRemarks: collected by Shuo Liu on 5 September 2018; **Record Level:** basisOfRecord: preserved specime
- **Type status:** Other material. **Occurrence:** catalogNumber: KIZ 059176; individualCount: 1; sex: female; lifeStage: adult; **Location:** country: China; stateProvince: Yunnan; locality: Nabang Town, Yingjiang County, Dehong Prefecture; verbatimElevation: 320 m; verbatimCoordinates: 24°45'47"N 97°34'15"E; **Event:** eventRemarks: collected by Shuo Liu on 5 September 2018; **Record Level:** basisOfRecord: preserved specime
- **Type status:** Other material. **Occurrence:** catalogNumber: KIZ20209131; individualCount: 1; lifeStage: juvenile; **Location:** country: China; stateProvince: Yunnan; locality: Xueli Village, Taiping Town, Yingjiang County, Dehong Prefecture; verbatimElevation: 350 m; verbatimCoordinates: 24°26'32"N, 97°33'4"E; **Event:** eventRemarks: collected by Shuo Liu on 13 September 2020; **Record Level:** basisOfRecord: preserved specime
- **Type status:** Other material. **Occurrence:** catalogNumber: KIZ20209132; individualCount: 1; lifeStage: juvenile; **Location:** country: China; stateProvince: Yunnan; locality: Xueli Village, Taiping Town, Yingjiang County, Dehong Prefecture; verbatimElevation: 350 m; verbatimCoordinates: 24°26'32"N, 97°33'4"E; **Event:** eventRemarks: collected by Shuo Liu on 13 September 2020; **Record Level:** basisOfRecord: preserved specime
- **Type status:** Other material. **Occurrence:** catalogNumber: KIZ20209133; individualCount: 1; lifeStage: juvenile; **Location:** country: China; stateProvince: Yunnan; locality: Xueli Village, Taiping Town, Yingjiang County, Dehong Prefecture; verbatimElevation: 350 m; verbatimCoordinates: 24°26'32"N, 97°33'4"E; **Event:** eventRemarks: collected by Shuo Liu on 13 September 2020; **Record Level:** basisOfRecord: preserved specime
- **Type status:** Other material. **Occurrence:** catalogNumber: KIZ20209134; individualCount: 1; lifeStage: juvenile; **Location:** country: China; stateProvince: Yunnan; locality: Xueli Village, Taiping Town, Yingjiang County, Dehong Prefecture; verbatimElevation: 350 m; verbatimCoordinates: 24°26'32"N, 97°33'4"E; **Event:** eventRemarks: collected by Shuo Liu on 13 September 2020; **Record Level:** basisOfRecord: preserved specime
- **Type status:** Other material. **Occurrence:** catalogNumber: KIZ20209135; individualCount: 1; lifeStage: juvenile; **Location:** country: China; stateProvince: Yunnan; locality: Xueli Village, Taiping Town, Yingjiang County, Dehong Prefecture; verbatimElevation: 350 m; verbatimCoordinates: 24°26'32"N, 97°33'4"E; **Event:** eventRemarks: collected by Shuo Liu on 13 September 2020; **Record Level:** basisOfRecord: preserved specime

#### Description

##### Description of the two adult specimens (KIZ 059299 and KIZ 059167)

Morphometric and meristic data are presented in Table 2. Male body large (SVL 116.4 mm), female body relatively small (SVL 97.2 mm). Tail relatively short (TaiL/SVL 2.10 in male and 1.95 in female), extremities relatively short and robust. Head large, distinct from the neck and lateral sides flat. Posterior parts of jaw angle slightly swollen in male, not swollen in female. Snout-tip blunt. Nostril in a single scale, separated from the labial scale by one or two scales. Rostral and mental scales small. Canthus rostralis sharp and straight from the nostril to the posterior part of the eye, including 6–7 scales between the nostril and the eye and 8–9 supraciliary scales. Ten supralabial scales, 9 infralabial scales. Tympanum distinct. Two short separated spines on each side of the upper head above the tympanum and two shorter spines beside each of them. Scales on chin and throat keeled. Nuchal crest with 10–11 scales, dorsal crest with 35–39 scales. Nuchal and dorsal crest well developed and high in male, composed of erected compressed scales, directed posteriorly, highest above the insertion of the front limbs and gradually decreasing towards the tail. Nuchal crest developed in female, composed of erected compressed scales, dorsal crest poorly developed in female. Oblique fold of skin in front of forelimb insertion distinct, covered with small granular dark scales. Dorsal scales feebly keeled, pointing upwards and backwards. Ventral scales parallel and strongly keeled. Caudal scales keeled, directed backwards. Subcaudal scales parallel and strongly keeled.

##### Colouration

**Figure 2.**
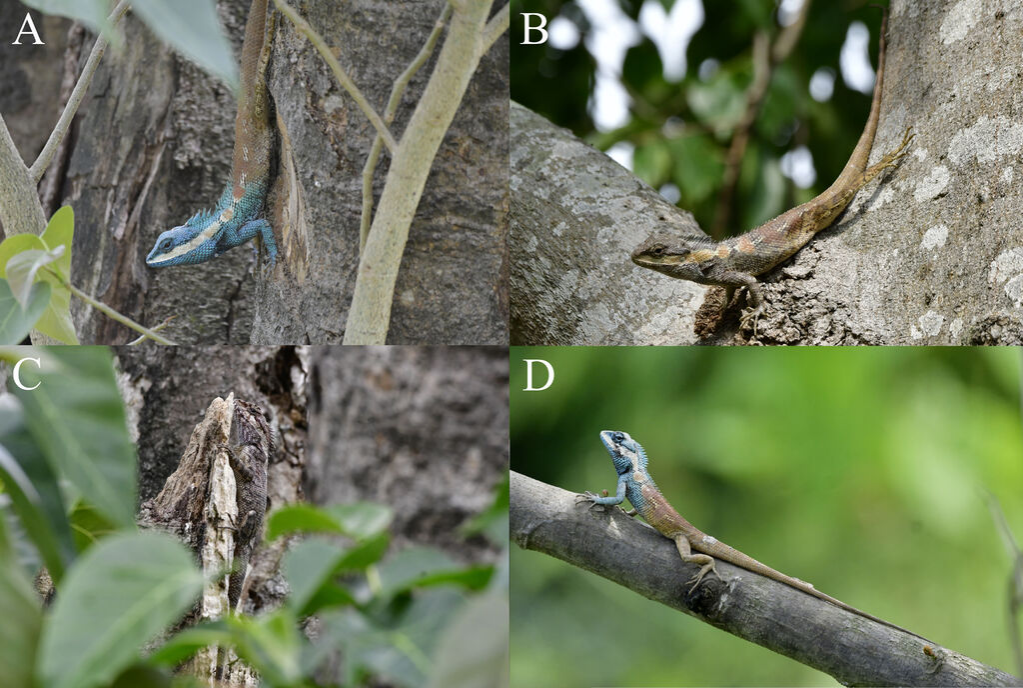
*Calotesvindumbarbatus* from Tongbiguan Nature Reserve, western Yunnan, China, in life. **A** an adult male in bright colouration; **B** an adult male in sombre colouration; **C** an adult female; **D** a subadult male with smaller body size and low nuchal and dorsal crest.

This species has a very strong ability to change the body colouration (Fig. 2). Usually, the head, forelimbs and anterior half of the body of adult males are blue, a white stripe, as high as the tympanum, is present from between nostril and orbit along the upper lip and the tympanum to the insertion of the front limb. The stripe is followed by 4–5 white blotches and there are thinner white stripes between each blotch. Gradually increasing brown blotches are present just on each white blotch, the first two are smaller than the white blotches and the rest almost covered the whole of the white blotches. The posterior half of the body, hind limbs and tail almost uniform brown. When they were disturbed or the environment changed etc., they can change their body colouration. The head, forelimbs and anterior half of the body may become brownish-grey, the white stripe become brownish-yellow, the posterior half of the body, hindlimbs and tail are still brown, but darker.

The colourations of adult females are relatively dim. Usually, the ground colour of females is purple brown, there are dark longitudinal stripes on the chin region and dark reticulate stripes on the back and there are radial dark stripes around the eyes, the white stripe along the upper lip and the dorsolateral blotches are more indistinct. However, the females can also change their body colouration. The stripes on the back, chin region and around the eyes may become indistinct. The head, forelimbs and anterior half of the body may become bluish, the white stripe and dorsolateral blotches may become more distinct. However, the colourations of females are still not as bright as those of males.

#### Diagnosis

##### Revised diagnosis

A medium-sized *Calotes* species of the complex, males with a known maximum SVL of 116.4 mm, females with a SVL of 97.2 mm. Tail length short, approximately twice the length of SVL. It can be distinguished from the other species of the complex by the combination of the following characters: 1) head and body robust, posterior parts of jaw angle slightly swollen in adult males, not swollen in female; 2) dorsal scales large, feebly keeled, pointing upwards and backwards, ventral scales small, parallel and strongly keeled; 3) body scales arranged in 50–56 rows around mid-body; 4) 18–22 lamellae on the fourth finger and 22–27 lamellae on the fourth toe; 5) 40–49 vertebral scales, nuchal crest developed in adults, dorsal crest developed in adult males, but undeveloped in females; 6) two short separated spines on each side above the tympanum and two shorter spines beside each of them; 7) oblique fold of skin in front of forelimb insertion distinct, covered with small granular dark scales; 8) the head, forelimbs and anterior half of the body blue in adult males, white stripe along the upper lip present, the posterior half of the body, hindlimbs and tail almost uniform brown; 9) 4–5 white blotches on each side of lateral body, gradually increasing brown blotches are present just on each white blotch, the first two are smaller than the white blotches and the rest almost covering the whole of the white blotches; 10) the colourations of adult females similar to those of males, but relatively dim; 11) nuchal and dorsal crest undeveloped in juveniles, the white stripe along the upper lip distinct, but the dorsolateral blotches indistinct and dark reticulate pattern present on the back in juveniles.

#### Ecology

##### Ecological notes

**Figure 3.**
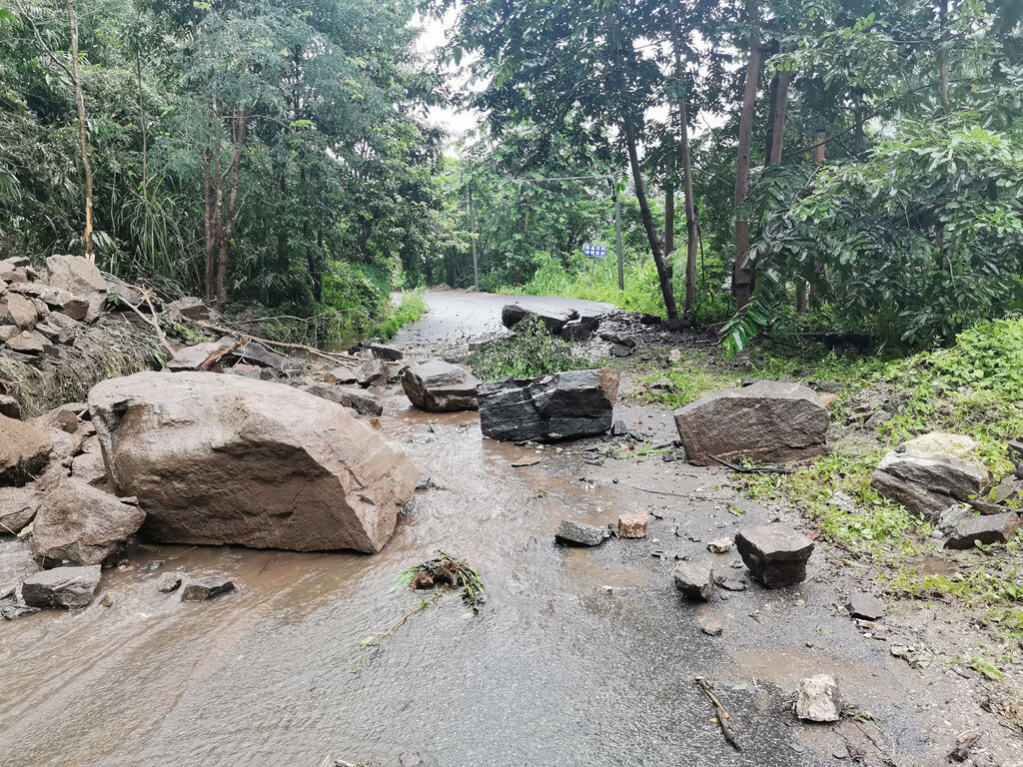
Habitat of *Calotesvindumbarbatus* at Xueli Village, Taiping Town, Yingjiang County, Dehong Prefecture, Yunnan Province, China.

The specimens were found on the trunks beside roads during the day (Fig. 3) and were found on the branches at night. Two other species of *Calotes*, *C.irawadi* Zug, Brown, Schulte & Vindum, 2006 and *C.emma* Gray, 1845 were observed to be sympatric with this species.

## Analysis

**Figure 4.**
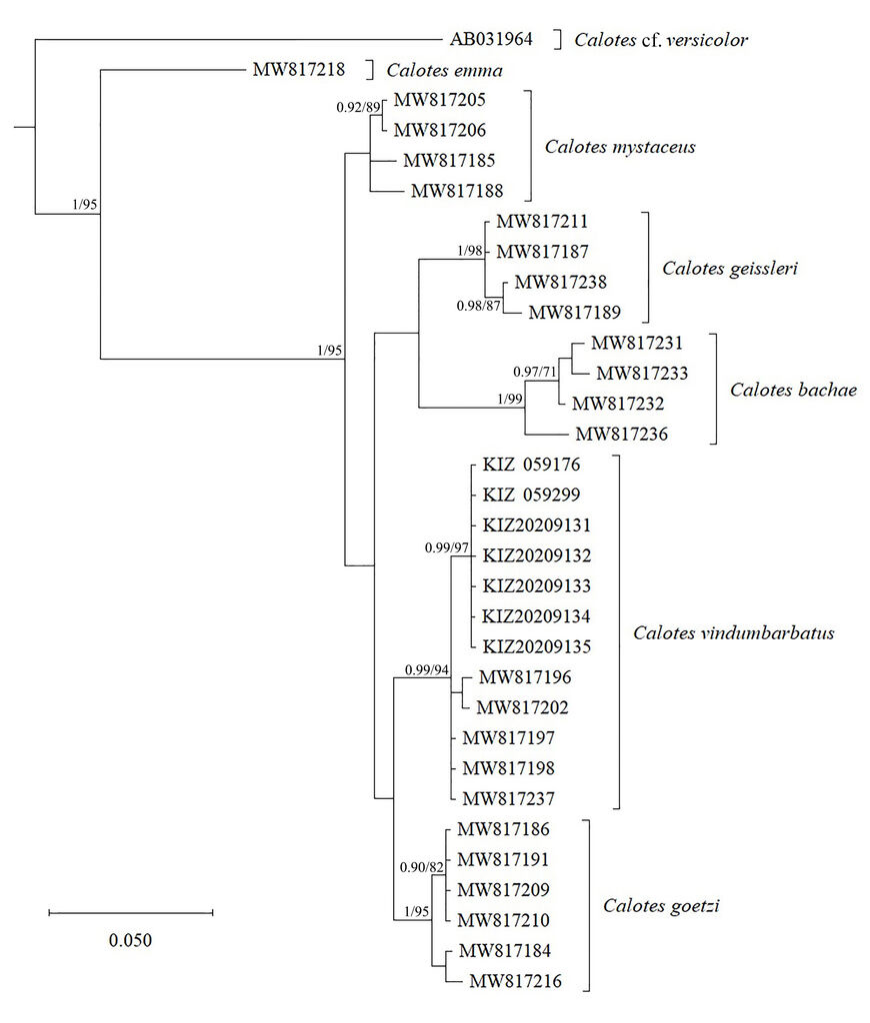
Bayesian Inference tree, based on mitochondrial 12S rRNA sequences. Numbers before slashes indicate Bayesian posterior probabilities (values below 0.90 are not shown) and numbers after slashes indicate bootstrap support for Maximum Likelihood analyses (values below 70 are not shown).

BI and ML analyses showed consistent topology (Fig. 4). The seven specimens collected from Tongbiguan Natural Reserve, western Yunnan, China, were homogeneous and clustered with the type specimens of *Calotesvindumbarbatus* from Myanmar with strong support. The genetic distance (uncorrected p-distance) between the specimens from China and the type specimens of *C.vindumbarbatus* from Myanmar was only 0.9% (Table 3). Therefore, we considered these specimens from China belong to *C.vindumbarbatus*.

According to Wagner et al. (2021), *Calotesvindumbarbatus* has a small body size (maximum SVL 77 mm), low nuchal and dorsal crest, no brownish dorsolateral blotches. Some of the newly-collected specimens of *C.vindumbarbatus* from western Yunnan agree well with these diagnoses; however, some of the newly-collected specimens of *C.vindumbarbatus* from western Yunnan have much larger body sizes (maximum SVL 116.4 mm), much longer nuchal and dorsal crest and obvious brown dorsolateral blotches. Therefore, we consider that the specimens of *C.vindumbarbatus* in Wagner et al. (2021) are juveniles rather than adults and the description and diagnosis of this species in Wagner et al. (2021) are only based on juveniles. Herein, we provide supplementary description of adults, based on the newly-collected specimens and revised diagnosis of this species.

## Discussion

**Figure 5.**
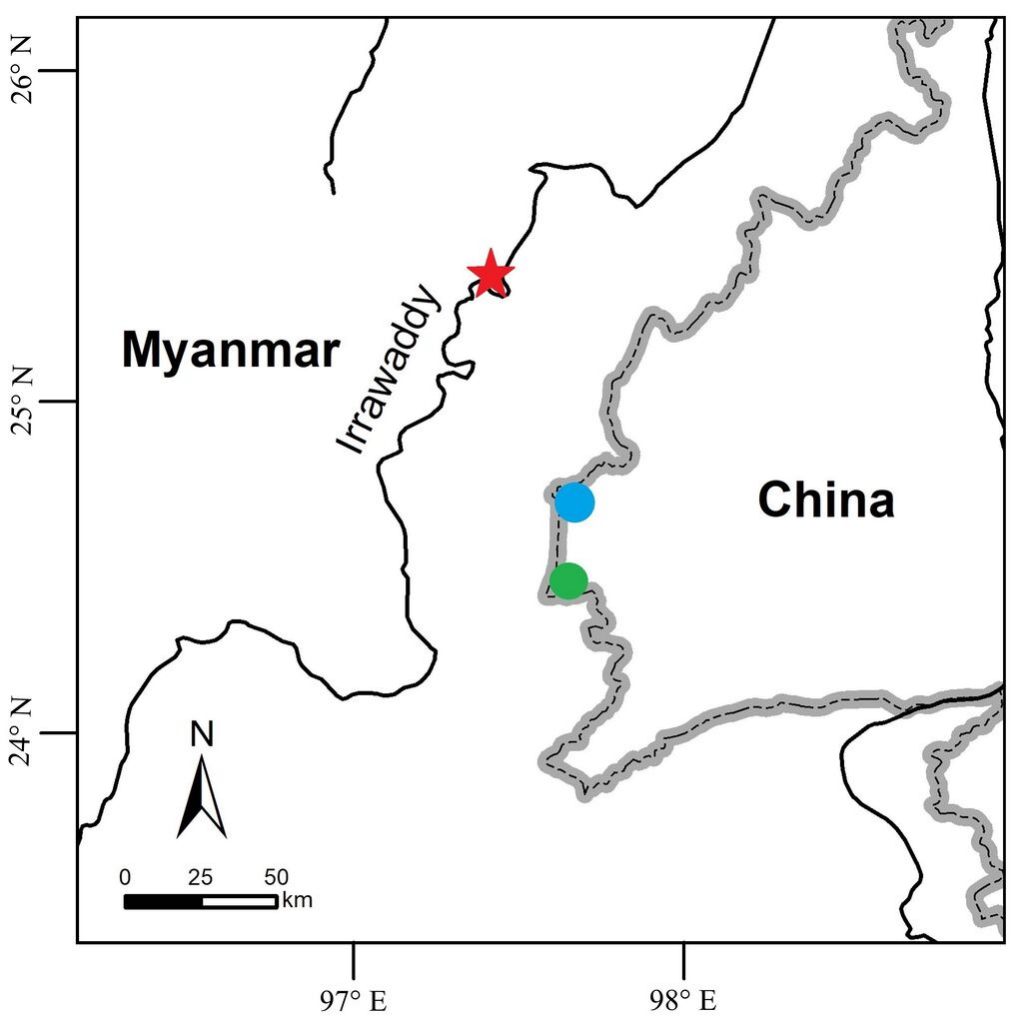
Collection sites of *Calotesvindumbarbatus* in China (green dot and blue dot) and the type locality (red star) of *C.vindumbarbatus* in Myanmar.

*Calotesvindumbarbatus* was known previously only from northern Myanmar (Wagner et al. 2021). This is the first record of *C.vindumbarbatus* from China and from outside of Myanmar. The new localities in China are approximately 75–100 km away from the type locality in Myanmar (Fig. 5).

Previously, *Calotesmystaceus* was considered to be widely distributed from India, Myanmar, China, Laos, Cambodia, Thailand and Vietnam (Bain and Hurley 2011, Hartmann et al. 2013, Chan-Ard et al. 2015, Das 2015, Pham et al. 2018). Wagner et al. (2021) restricted *C.mystaceus* to southern coastal Myanmar. Therefore, *C.mystaceus* is not distributed in China. According to Wagner et al. (2021) and this study, the species, previously confused with *C.mystaceus* in China, actually refer to *C.goetzi* and *C.vindumbarbatus*.

Gowande et al. (2021) restricted *Calotesversicolor* (Daudin, 1802) to parts of southern and eastern India. Therefore, *C.versicolor* is also not distributed in China. Liu et al. (2021) recorded *C.irawadi* in China. In conclusion, there are seven species of *Calotes* distributed in China to date, namely: *C.emma* Gray, 1845; *C.goetzi* Wagner, Ihlow, Hartmann, Flecks, Schmitz & Böhme, 2021; *C.irawadi* Zug, Brown, Schulte & Vindum, 2006; *C.jerdoni* Günther, 1870; *C.medogensis* Zhao & Li, 1984; *C.paulus* (Smith, 1935); and *C.vindumbarbatus* Wagner, Ihlow, Hartmann, Flecks, Schmitz & Böhme, 2021.

The fauna of the agamids in China was analysed 10 years ago (Ananjeva et al. 2011). Thereafter, the research on the cryptic diversity of agamids in China has made continuous progress. Wang et al. (2020b) compiled the checklists of amphibians and reptiles of China as at the end of 2019. Compared with ten years ago, Wang et al. (2020b) recorded 66 species of the agamids in China, 17 species more than Ananjeva et al. (2011). There are minor changes at the genus level, *Oriocalotes* Günther, 1864 was abolished and *Diploderma* Hallowell, 1861 was resurrected; therefore, the agamids in China still consist of 12 genera (Wang et al. 2020b). In the past two years, some more new species and new records of the agamids from China have been described (e.g. Liu et al. 2020a, Liu et al. 2020b, Wang et al. 2020a, Liu et al. 2021, Wang et al. 2021). However, the diversity of the agamids in China is far from clear and more research in this field is needed.

## Supplementary Material

XML Treatment for Calotes vindumbarbatus

## Figures and Tables

**Table 1.** Sequences (12S rRNA) used for phylogenetic analysis.

Species	Voucher no.	Locality	GenBank no.	Reference
*Calotesbachae*	ZFMK 88935 (holotype)	Vietnam: Dong Nai: Cat Tien National Park	MW817231	Wagner et al. (2021)
*Calotesbachae*	ZFMK 92028	Vietnam: Cao Bang: Trung Khanh	MW817232	Wagner et al. (2021)
*Calotesbachae*	ZFMK 96231	Vietnam	MW817236	Wagner et al. (2021)
*Calotesbachae*	MW817236	Cambodia: Ratanakiri: Banlung	MW817233	Wagner et al. (2021)
*Calotesgeissleri*	CAS 215539 (holotype)	Myanmar: Sagaing: Alaungdaw Kathapa National Park	MW817189	Wagner et al. (2021)
*Calotesgeissleri*	CAS 210270 (paratype)	Myanmar: Sagaing: Alaungdaw Kathapa National Park	MW817187	Wagner et al. (2021)
*Calotesgeissleri*	ZFMK 97991 (paratype)	Myanmar: Chin: Falam: Simggial Village	MW817238	Wagner et al. (2021)
*Calotesgeissleri*	CAS 243028 (paratype)	Myanmar: Magway: Gangaw: Gangaw: Mauk Village	MW817211	Wagner et al. (2021)
*Calotesgoetzi*	CAS 242463	China: Yunnan: Baoshan: Longling	MW817210	Wagner et al. (2021)
*Calotesgoetzi*	CAS 242457	China: Yunnan: Baoshan: Longyang	MW817209	Wagner et al. (2021)
*Calotesgoetzi*	CAS 228144	China: Yunnan: Nujiang	MW817191	Wagner et al. (2021)
*Calotesgoetzi*	CAS 207489	China: Yunnan: Nujiang	MW817186	Wagner et al. (2021)
*Calotesgoetzi*	CAS 204849	Myanmar: Mandalay	MW817184	Wagner et al. (2021)
*Calotesgoetzi*	NME R 0584/09a	Thailand: Chiang Mai	MW817216	Wagner et al. (2021)
*Calotesmystaceus*	CAS 240296	Myanmar: Mon: Kyaikhto	MW817206	Wagner et al. (2021)
*Calotesmystaceus*	CAS 206548	Myanmar: Yangon: Letpein Village	MW817185	Wagner et al. (2021)
*Calotesmystaceus*	CAS 240287	Myanmar: Mon: Kyaikhto	MW817205	Wagner et al. (2021)
*Calotesmystaceus*	CAS 213300	Myanmar: Yangon: Hlawga National Park: Mingalardon	MW817188	Wagner et al. (2021)
*Calotesvindumbarbatus*	CAS 232388 (holotype)	Myanmar: Kachin: Myitkyina: Gat Shang Yang Village	MW817198	Wagner et al. (2021)
*Calotesvindumbarbatus*	CAS 232387 (paratype)	Myanmar: Kachin: Myitkyina: Gat Shang Yang Village	MW817197	Wagner et al. (2021)
*Calotesvindumbarbatus*	ZFMK 97990 (paratype)	Myanmar: Kachin: Myitkyina: Gat Shang Yang Village	MW817237	Wagner et al. (2021)
*Calotesvindumbarbatus*	CAS 239206 (paratype)	Myanmar: Sagaing: Hkanti: Hkanti: Linpha Village	MW817202	Wagner et al. (2021)
*Calotesvindumbarbatus*	CAS 232247 (paratype)	Myanmar: Sagaing: Homalin: N of Swekawngaw	MW817196	Wagner et al. (2021)
*Calotesvindumbarbatus*	KIZ20209131	China: Yunnan: Dehong: Yingjiang: Taiping Town	OM418450	This study
*Calotesvindumbarbatus*	KIZ20209132	China: Yunnan: Dehong: Yingjiang: Taiping Town	OM418451	This study
*Calotesvindumbarbatus*	KIZ20209133	China: Yunnan: Dehong: Yingjiang: Taiping Town	OM418452	This study
*Calotesvindumbarbatus*	KIZ20209134	China: Yunnan: Dehong: Yingjiang: Taiping Town	OM418453	This study
*Calotesvindumbarbatus*	KIZ20209135	China: Yunnan: Dehong: Yingjiang: Taiping Town	OM418454	This study
*Calotesvindumbarbatus*	KIZ 059176	China: Yunnan: Dehong: Yingjiang: Nabang Town	OM418455	This study
*Calotesvindumbarbatus*	KIZ 059299	China: Yunnan: Dehong: Yingjiang: Nabang Town	OM418456	This study
*Calotesemma*	NME R 0590/09	Laos: Phongsali	MW817218	Wagner et al. (2021)
Calotescf.versicolor	35570	Thailand: Ko Chang	AB031964	Honda et al. (2000)

**Table 2.** Measurements (in mm) and scalation data for the specimens of *Calotesvindumbarbatus* collected from China. For character abbreviations, see material and methods. Paired meristic characters were made on the left side.

	KIZ 059299 Adult male	KIZ 059176 Adult female	KIZ20209131 Juvenile	KIZ20209132 Juvenile	KIZ20209133 Juvenile	KIZ20209134 Juvenile	KIZ20209135 Juvenile
SVL	116.4	97.2	79.5	67.4	72.5	52.6	45.0
EyeEar	8.6	6.1	5.2	4.3	4.9	3.7	3.3
HeadH	18.7	13.9	13.3	11.4	12.1	9.3	8.4
HeadL	29.8	23.5	19.7	17.3	18.7	14.1	11.9
HeadW	23.8	18.2	16.7	14.1	15.1	11.6	9.9
Interorb	14.8	12.2	10.4	9.0	9.6	7.2	5.8
JawW	22.0	18.2	16.7	14.1	15.1	11.6	9.9
NarEye	8.0	5.6	5.7	4.7	4.7	3.3	2.8
SnEye	13.0	10.1	9.6	7.7	8.6	6.1	5.1
SnW	7.4	6.7	5.9	4.9	5.5	3.7	3.7
4FingL	13.6	10.9	10.7	9.5	9.9	6.8	6.4
4ToeL	17.4	15.9	16.9	13.9	11.3	9.7	8.6
CrusL	24.1	20.3	18.1	15.6	16.9	12.7	10.1
ForefL	20.2	16.9	16.3	14.7	14.8	11.0	9.8
HindfL	32.3	28.4	28.9	24.3	22.7	17.7	15.4
LoArmL	18.9	16.3	13.8	12.2	13.1	9.2	7.4
SnForeL	43.9	35.1	29.6	25.3	27.2	20.7	17.0
TailH	14.4	9.4	9.8	7.4	9.1	5.6	4.8
TailL	244.0	189.5	179.5	144.0	154.5	106.0	88.5
TailW	12.7	9.4	8.8	7.2	7.7	5.6	4.6
TrunkL	52.9	45.0	36.6	30.3	32.4	22.1	20.2
UparmL	23.6	19.1	16.4	14.7	14.8	10.6	9.0
UplegL	23.9	20.8	18.7	15.4	17.7	12.4	9.9
CanthR	8	9	10	9	9	9	10
Eyelid	13	12	11	13	11	11	11
HeadSLn	15	16	16	17	17	18	17
HeadSTr	14	13	15	17	14	14	16
Inflab	9	9	10	10	10	11	11
Sns	7	6	7	8	7	7	7
Suplab	10	10	9	10	9	11	11
4FingLm	19	19	20	20	18	21	22
4ToeLm	24	23	24	27	22	25	24
VertS	46	49	48	47	46	49	48
MidbS	53	53	56	52	50	53	52

**Table 3.** Mean uncorrected p-distances (%), based on 12S rRNA sequences.

	1	2	3	4	5	6	7
1 *Calotesbachae*							
2 *Calotesgeissleri*	6.3						
3 *Calotesgoetzi*	6.7	4.9					
4 *Calotesmystaceus*	6.3	4.6	3.2				
5 *Calotesvindumbarbatus* (China)	6.8	5.2	3.7	4.5			
6 *Calotesvindumbarbatus* (Myanmar)	6.5	5.0	3.5	4.2	0.9		
7 *Calotesemma*	13.5	13.0	11.8	10.8	11.6	12.2	
8 Calotescf.versicolor	19.2	19.6	18.0	19.2	19.0	18.4	16.7
